# Infratentorial Stereotactic Biopsy of Brainstem and Cerebellar Lesions

**DOI:** 10.3390/brainsci11111432

**Published:** 2021-10-28

**Authors:** Jacek Furtak, Paulina Śledzińska, Marek G. Bebyn, Tadeusz Szylberg, Stanisław Krajewski, Marcin Birski, Marek Harat

**Affiliations:** 1 Department of Neurosurgery, 10th Military Research Hospital and Polyclinic, 85-681 Bydgoszcz, Poland; krajewskirehabilitacja@wp.pl (S.K.); mbirski@poczta.fm (M.B.); harat@10wsk.mil.pl (M.H.); 2 Franciszek Lukaszczyk Oncology Center, Department of Neurooncology and Radiosurgery, 85-796 Bydgoszcz, Poland; 3 Faculty of Medicine, Collegium Medicum, Nicolaus Copernicus University, 85-067 Bydgoszcz, Poland; paula.sledzinska@gmail.com; 4 Faculty of Medicine, Medical University of Gdańsk, 80-210 Gdańsk, Poland; marek.bebyn@gmail.com; 5 Department of Pathomorphology, 10th Military Research Hospital, 85-681 Bydgoszcz, Poland; szylberg@10wsk.mil.pl; 6 Department of Physiotherapy, University of Bydgoszcz, 85-059 Bydgoszcz, Poland; 7 Department of Neurosurgery and Neurology, Collegium Medicum in Bydgoszcz, Nicolaus Copernicus University, 87-100 Toruń, Poland

**Keywords:** brainstem tumors, infratentorial approach, molecular analyses, procedural complications, stereotactic biopsy, *Toxoplasma gondii*

## Abstract

Stereotactic biopsy of posterior fossa lesions is often regarded as hazardous due to the critical structures in that area. Therefore, the aim of the study was to evaluate the diagnostic accuracy and safety of infratentorial stereotactic biopsy of brainstem or cerebellar lesions and its associations with other clinical, laboratory, and radiological parameters. From January 2000 to May 2021, 190 infratentorial stereotactic biopsies of posterior fossa tumors, including 108 biopsies of brainstem lesions, were performed. Moreover, 63 supratentorial biopsies of cerebral peduncle lesions were analyzed to compare the safety and efficacy of both approaches. Additionally, the presence of antibodies against *Toxoplasma gondii* and Epstein–Barr Virus (EBV) were documented in 67 and 66 patients, respectively, and magnetic resonance imaging (MRI) scans were evaluated in 114 patients. Only 4% of patients had minor complications and 1.5% had major complications, including one patient who died from intracranial bleeding. Nine (4.7%) biopsies were non-diagnostic. Isocitrate dehydrogenase 1 (*IDH1*) mutation, 1p/19q codeletion, and O6-methylguanine-DNA methyltransferase (*MGMT*) promoter methylation status were assessed in 29 patients, and were non-diagnostic in only 3 (10.3%) cases. Patients with high-grade gliomas (HGG) were more frequently seropositive for *T. gondii* than individuals with low-grade gliomas (LGG; *p* < 0.001). A total of 27% of HGG and 41% of LGG were non-enhancing on MRI. The infratentorial approach is generally safe and reliable for biopsy of brainstem and cerebellar lesions. In our study, the safety and efficacy of supratentorial biopsy of the cerebral peduncle and infratentorial biopsy of lesions below the cerebral peduncle were comparably high. Moreover, patients with HGG were more frequently seropositive for *T. gondii* than patients with LGG, and the relationship between toxoplasmosis and gliomagenesis requires further investigation.

## 1. Introduction

The biopsy of posterior fossa and brainstem tumors is often perceived as challenging. However, a significant number of brain tumors arise in this region and need a biopsy to guide clinical management [1,2]. A biopsy is indicated when surgical resection is not safely feasible e.g., due to tumor location or the patient’s compromised clinical status [3]. The main advantage of stereotactic biopsy is its low invasiveness and the ability to plan the biopsy trajectory to sample all relevant tumor sites, including the tumor infiltrating zone, contrast-enhancing locations, and radiographically suspected necrotic areas. Stereotactic biopsy’s goal is to gather reliable histological material in the most advantageous, quickest, and safest manner possible. Open surgery provides large amounts of tissue for diagnosis, but with a more random sampling technique and a higher risk of perioperative morbidity [4]. While several neuroimaging methods are now used in clinical practice, glioma classification and grading by magnetic resonance imaging (MRI) is <35% accurate [2]. Moreover, the fifth edition of the WHO Classification of Tumors of the Central Nervous System (WHO CNS5) 2021 places significant emphasis on molecular diagnostics for accurate classification [5], so tissue samples are required for state-of-the-art glioma stratification [6,7,8,9].

**Figure 1:**
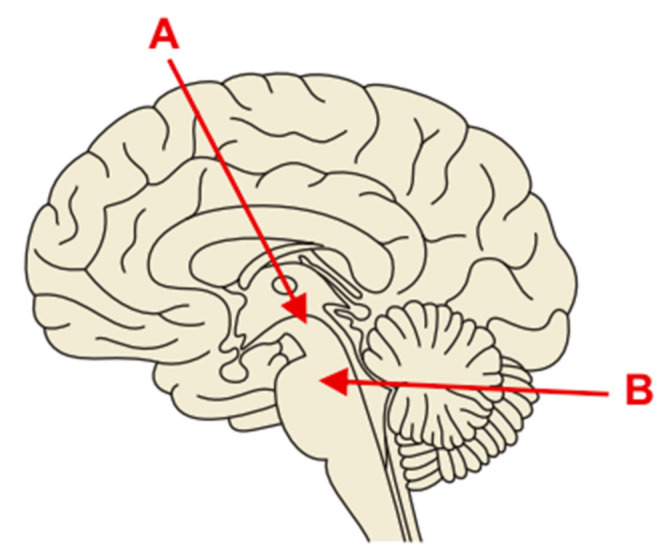
Scheme of the approaches of stereotactic biopsy. (**A**) The biopsy trajectory of the tumor located around the cerebral peduncles (supratentorial approach). (**B**) The biopsy trajectory of the brainstem tumors below the level of the peduncles (infratentorial approach).

Currently, material from CNS lesions can be obtained by stereotactic biopsy or biopsy with neuronavigation; open biopsy, especially of deep-seated structures, is now outdated [10,11,12]. Stereotactic biopsies of brainstem lesions can be performed in several different ways, depending on the lesion’s location. Brainstem tumors located within the cerebral peduncles are accessed supratentorially, while tumors located below the peduncles should be accessed only infratentorially [13,14,15] (see Figure 1).

However, there is only limited published data on clinical success rates and the diagnostic accuracy of infratentorial biopsy, despite the importance of this information to guide clinical practice. Therefore, we leveraged a large patient sample to study clinical and diagnostic outcomes of infratentorial biopsy and associations with other laboratory and radiological parameters.

## 2. Materials and Methods

One-hundred and ninety stereotactic infratentorial biopsies of brainstem and cerebellar malignancies were performed between January 2000 and May 2021 at the Neurosurgery Department, 10 Military Research Hospital in Bydgoszcz, Poland, accounting for 6.8% of all (2804 cases) stereotactic biopsies conducted at the hospital. The Ethics Committee of the Nicolaus Copernicus University, Collegium Medicum in Bydgoszcz, Poland approved the study protocol (KB 389/2021). Patient consent was not required because the study was a retrospective analysis of medical records.

**Figure 2:**
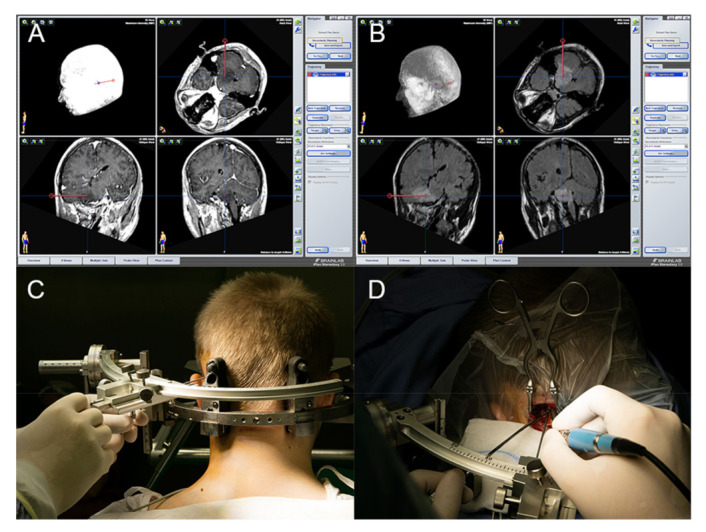
(**A**) T1-weighted image of a tumor located in the pons, cerebellar peduncle, and cerebellar hemisphere. (**B**) Fluid attenuated inversion recovery magnetic resonance imaging (FLAIR MRI) of a tumor located in the pons, cerebellar peduncle, and cerebellar hemisphere. (**C**) Patient with a stereotactic frame. (**D**) Suboccipital burr holes made using a high-speed drill.

Biopsy was performed on patients who could not safely undergo microsurgical excision due to the tumor’s location or the patient’s clinical condition. A frame-based method was used for the infratentorial approach (Figure 2). Biopsy was performed under local anesthesia, in a semi-sitting position, supported by MRI/CT fusion images and using the stereotactic system and software provided by Brainlab AG (Feldkirchen, Germany) (Figure 2A,B). From November 2011, the Leiblinger system by Inomed (Emmendingen, Germany) was used. Suboccipital burr holes were made using a high-speed drill (Figure 2C,D). The contrast-enhanced part of the lesion, or its center in cases of non-enhancing pathologies, was selected for tissue sampling. Tissue (usually between four and eight samples) was obtained using biopsy forceps. In many cases, a preliminary pathomorphological evaluation was conducted by a neuropathologist in the operating theater based on intraoperative methylene blue staining to ensure that the acquired material was representative of the lesion and to make an intraoperative diagnosis. Thereafter, tissue sections were subjected to detailed histopathological and molecular neuropathological examination based on CNS5 WHO 2021 criteria. All patients had a routine preoperative MRI and underwent a postoperative computed tomography (CT) scan for postoperative evaluation, irrespective of the clinical condition.

We performed a retrospective analysis of anonymized medical records. Age, gender, Karnofsky performance status (KPS), tumor location and features, histological diagnosis, complications, symptoms described by patients before biopsy, and blood levels of antibodies against *Toxoplasma gondii* and Epstein–Barr Virus (EBV) were tabulated and analyzed. In addition, where available, contrast-enhanced head MRIs were reviewed to see if the lesion was enhancing. Stereotactic biopsy samples obtained from 2015 were subjected to molecular testing including determination of isocitrate dehydrogenase (*IDH1*) mutation, 1p/19q codeletion, and O6-methylguanine-DNA methyltransferase (*MGMT*) promoter methylation status.

Additionally, we conducted a retrospective analysis of medical records of patients who had undergone supratentorial biopsy of cerebral peduncle lesions between January 2000 and May 2021 at the Neurosurgery Department, 10 Military Research Hospital in Bydgoszcz, Poland. The evaluation was performed to compare the efficacy and safety of supratentorial biopsy of tumors located within the cerebral peduncles and infratentorial biopsy of brainstem lesions located below cerebral peduncles.

The Shapiro–Wilk test was performed to test the normality of data. The Mann–Whitney and chi-squared tests were used to determine statistically significant differences between two groups of independent variables, depending on whether the data were continuous or categorical, respectively. The findings were measured using 95% confidence intervals (95% CI), and a *p*-value of 0.05 was considered statistically significant.

## 3. Results

Biopsies were performed in 96 women and 94 men aged between 14 and 77 (average age 41). Of 190 frame-based biopsies, only 9 (4.7%) were non-diagnostic. The most common diagnoses were astrocytoma grade 2 (35.8%) and astrocytoma grade 3 (19.5%). Almost 95% of infratentorial biopsies were without complications; 4% had minor and 1.5% had major complications, including one patient who died from intracranial bleeding (Table 1). Patients with and without complications related to a brainstem biopsy showed no significant differences in age, gender, diagnosis, location, or surgical approach.

**Figure 3:**
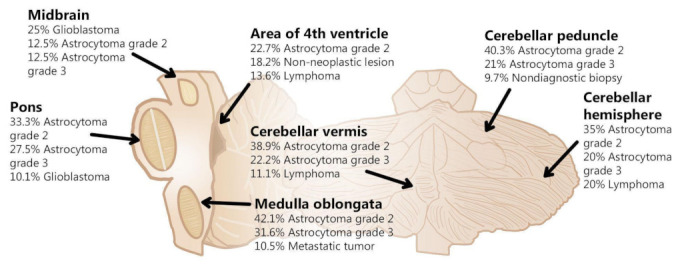
The frequency of diagnoses depending on lesion location.

The locations of the biopsied tumors are shown in Table 2. The proportions of tumors arising in the cerebellum, brainstem, and brainstem/cerebellum were approximately equal. The cerebellar peduncle was the most common location (26 tumors, 13.7%), followed by the cerebellar hemisphere (25, 13.2%) and pons (22, 11.6%). At most locations, the most frequent diagnosis was astrocytoma grade 2 and, in the cerebellar peduncle, nearly 10% of biopsies were non-diagnostic (Figure 3). Fifteen (7.9%) patients had multifocal tumors, none of which were metastatic, instead the most common diagnosis being lymphoma (*N* = 6) (Table 1). A secondary biopsy was conducted in seven individuals, six due to a previous non-diagnostic biopsy and one due to a suspicion of tumor progression.

Stereotactic biopsy material was also subjected to molecular testing in 29 patients, which was non-diagnostic in 3 (10.3%) patients. There were no 1p/19q co-deletions in any tumor, and most tumors were IDH-wildtype. High grade-gliomas (HGG, WHO grades 3 and 4) were more likely to have *MGMT* promoter methylation (Table 3), which was available in six patients: low in one patient, medium in four patients, and high in one patient. All were diagnosed with HGG.

**Figure 4:**
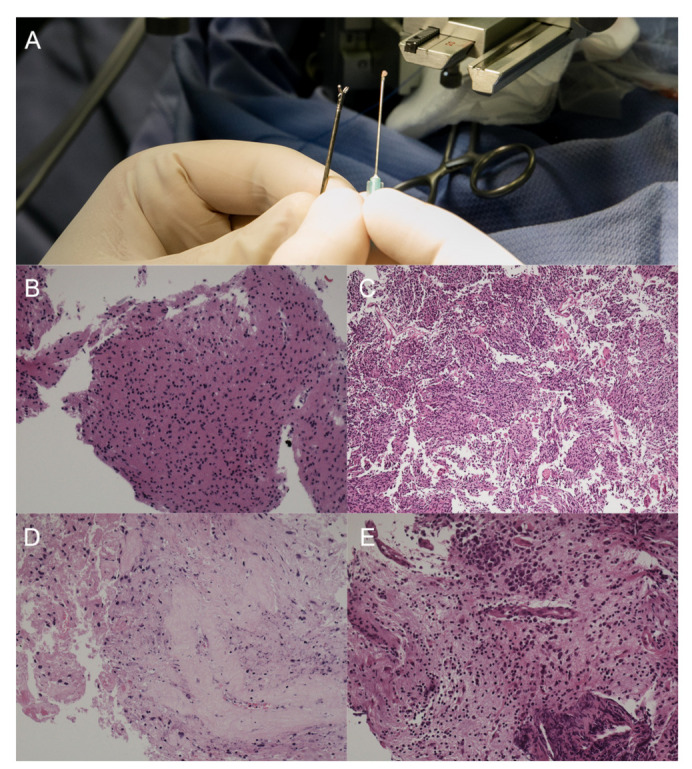
(**A**) The material obtained during a stereotactic biopsy. (**B**) Microscopic image of A2, H&E staining, ×200 magnification. (**C**) Microscopic image of A3, H&E staining, ×100 magnification. (**D**) Microscopic image of glioblastoma, H&E staining, ×200 magnification. (**E**) Microscopic image of diffuse large B-cell lymphoma (DLBCL), H&E staining, ×200 magnification.

A pathologist was present for 187 out of 190 biopsies. The material obtained during stereotactic biopsy, examples of intraoperative microscopic smears, and final photomicrographs of hematoxylin and eosin stained sections are presented in Figure 4. Patients with medulloblastoma or pilocytic astrocytoma had the highest KPS, while those with metastatic tumors and posterior fossa ependymoma grade 3 had the lowest. There was no significant relationship between diagnosis and gender (Table S1).

Serum antibodies against EBV were available for analysis in 66 patients and against *T. gondii* in 67 patients (Table S2). Antibodies to EBV were found in most patients (86.4%), regardless of the diagnosis. EBV seropositivity was observed in 92% of lower-grade glioma (LGG) (WHO grades 1 and 2) patients and 87% of HGG patients.

Serum antibody positivity for *T. gondii* was more variable (Table S2), but patients with LGG were more likely to be *T. gondii* antibody negative, while patients with HGG were more likely to be seropositive (*p* < 0.001) (Table 4). There was no statistically significant association between *T. gondii* IgG antibody titer and tumor malignancy.

One hundred and fourteen contrast-enhanced head MRIs were assessed to establish whether the lesions were enhancing. There was a statistically significant association between enhancement and tumor grade: HGG were more often contrast-enhancing than LGG (Table 4). However, 27% of HGG did not enhance and 41% of LGG enhanced. Patients with MRI-enhancing tumors were older (median = 46.5, *N* = 66) than those with non-enhancing MRI tumors (median = 36, *N* = 48) (Mann–Whitney test, *p* = 0.016).

Supratentorial biopsies of cerebral peduncles were performed in 23 women and 40 men aged between 20 and 78. Only 2 (3.2%) of 63 supratentorial stereotactic biopsies were non-diagnostic. The most common diagnoses were astrocytoma grade 3 (22.2%), followed by glioblastoma and lymphoma (17.5% each). Four (6.3%) patients had multifocal tumors, only one of which was metastatic. Biopsy material obtained through a supratentorial approach was also subjected to molecular testing in six cases, which was non-diagnostic only in one patient (see Table S3). About 98% of supratentorial biopsies of cerebral peduncles’ tumors were without complications. The comparison of infratentorial and supratentorial biopsy is presented in Table 5.

## 4. Discussion

This study is one of the largest to assess patients undergoing infratentorial biopsy of tumors of the brainstem and cerebellum. Our findings demonstrate that infratentorial biopsy is over 95% accurate and complication-free in 94.5% of patients. Moreover, the genetic profile of gliomas may be established with 89% accuracy using biopsy samples. In our study, the safety and efficacy of supratentorial biopsy of the cerebral peduncle and infratentorial biopsy of lesions below the cerebral peduncle were comparably high. Based on the results of our research, we confirm that the cerebral peduncle could serve as a dividing line between supratentorial and infratentorial approaches to brainstem tumor biopsy, which is in line with previous research [14]. By choosing the optimal approach, the trajectory can be shortened and critical structures omitted, which significantly reduces the risk of complications. Moreover, the high diagnostic yield by stereotactic biopsy was possible due to close cooperation between neurosurgeon and pathologist during surgery. So-called “empty” biopsies, taken without the presence of a pathologist and lacking preliminary intraoperative evaluation, increase the chance of a non-diagnostic biopsy [16], for example, when an area of necrosis is aspirated to yield a non-diagnostic biopsy. In our sample, we found that the largest percentage of non-diagnostic biopsies were from tumors occupying the cerebellar peduncle. This might be because not only was this the most common location for tumors but also because most needle pathways to brainstem tumors have passed through the middle cerebellar peduncle.

We detected a statistically significant association between the presence of *T. gondii* antibodies and glioma grade: patients with LGG were more likely to be Toxoplasma-negative and patients with HGG seropositive. To our best knowledge, this is a novel finding. It is possible that prior or hidden *T. gondii* infection may result in a more severe glioma course.

Surgery is generally beneficial only in the case of localized, exophytic, or cervicomedullary malignancies. In comparison, diffuse brainstem gliomas are considered unresectable lesions [17]. Stereotactic biopsy is typically performed when the tumor is inoperable. However, even if the tumor is operable, the risk of persistent neurological deficits due to surgery is 20–30% [18,19].

The reported effectiveness of brainstem tumor biopsy ranges from 87% to 100% [2,11,12,13,14,20,21,22,23], consistent with our findings, and the risk of complications varies from 0% to 11% [2,8,14,22,23,24]; our complication rate was ~4%. Tilgner et al. reported an intraoperative diagnostic accuracy of 90.3% [25], similar to our results. Stereotactic biopsies for intrinsic brainstem lesions were as safe and effective as biopsy of lesions in the supratentorial compartment [12]. Moreover, we previously reported that the initial histological findings obtained by stereotactic biopsy were the same as for open surgery [26]. Ramakonar et al. argued that even if the biopsy material was non-diagnostic in histological evaluations, molecular testing should be performed, since mutations in *IDH1* and *TERT* may still be detected [27]. Fritsch et al. associated a high diagnostic yield with the number of systematically obtained samples per lesion, with an increased number of biopsies not leading to an increase in complications [28]. The risk of complications can be reduced by irrigating the site repeatedly with 0.1–0.2 mL saline using a thin plastic tube until the fluid does not contain bloody fluids to ensure hemostasis [29]. Moreover, it appears reasonable to discharge patients the same day or within one day of stereotactic biopsy if the postoperative CT shows no complication [28].

Diagnosis of toxoplasmosis is primarily based on serological tests that detect *T. gondii*-specific IgG and IgM antibodies. Although the parasite forms cysts preferentially in the brain and a variety of brain cells, including astrocytes and neurons, can be infected, the relationship between *T. gondii* infection and brain tumors is scarcely described in the literature [30]. Schuman et al. showed that astrocytoma patients were significantly more likely than controls to have antibodies to *T. gondii* [20], and there is some prospective evidence of an association between *T. gondii* infection and risk of glioma [31]. Moreover, in epidemiological studies, *T. gondii* seropositivity among brain tumor patients (18.3%) was significantly (*p* < 0.05) higher than that of healthy controls (8.6%) [32]. In France, brain tumor mortality rates were positively associated with *Toxoplasma gondii* [33]. Conversely, in an Australian case-control study, Ryan et al. failed to detect an association between antibody positivity to *T. gondii* and risk of glioma [34]. Therefore, while there is some evidence of an epidemiological association between brain tumor and *T. gondii* seropositivity, this is the first study to examine *T. gondii* seroprevalence in patients with specific glioma subtypes.

Recent studies have shown that *T. gondii* activates the epidermal growth factor receptor (*EGFR*) pathway during invasion, which allows *T. gondii* to survive within host cells by avoiding autophagy-dependent lysosomal degradation [35,36]. Transgenic mice expressing a dominant negative *EGFR* in endothelial cells (to inhibit *EGFR* signaling) had a diminished parasite load and histopathological evidence of brain and retina involvement after *T. gondii* infection [37]. In WHO CNS5, *EGFR* gene amplification is a criterion to upgrade IDH-wildtype diffuse astrocytic tumors in adults to glioblastoma, IDH-wildtype. We are the first to demonstrate a statistical relationship between seropositivity and higher glioma grade in our cohort, which might support a mechanistic relationship between *T. gondii* infection and more malignant gliomas through *EGFR* pathway activation.

Multifocal lesions on MRI are frequently diagnosed as metastasis and are an indication to look for a primary site. However, we found no cases of disseminated disease, and care should be taken not to assume that every multifocal lesion is definitely a metastasis. Furthermore, 41% of LGG showed contrast enhancement and almost a third of HGG did not. Similar to our results, Ginsberg et al. reported that LGG accounted for 60% of non-enhancing brain neoplasms, but 40% of their non-enhancing lesions were classified as HGG [38]. Pallud et al. reviewed 927 histologically-proven WHO grade 2 gliomas, and 84.1% of them were non-enhancing on MRI [39]. While tumor enhancement is a valuable diagnostic clue it should not be used to distinguish malignant from benign tumors, since absence of contrast enhancement is neither a sensitive nor a specific sign of low-grade neoplasms.

Our study has several limitations. This was a retrospective, non-randomized study. Symptoms were not described in all patients, and serum antibodies against EBV and *T. gondii* were only available for investigation in 66 patients and 67 patients, respectively, due to testing only starting in 2015. The presence of contrast enhancement was not examined in 75 patients due to a lack of access to MRI exams before 2007 due to technical problems. Furthermore, we were unable to determine the precise site of tumors in those patients from imaging and relied only on information from the medical records. Molecular analyses were similarly only available for a limited number of patients, since this service started in 2016 after the introduction of the previous WHO classification.

Further studies would be helpful to confirm the efficacy of infratentorial biopsies for acquiring tissue for genetic testing. Despite posterior fossa tumor biopsy appearing to be safe, there always remains a risk of complications, and each complicated case requires detailed evaluation. Moreover, a larger group of patients is required to confirm the relationship between the presence of antibodies against *T. gondii* and an increased risk of developing HGG. Establishing a causal link between *T. gondii* infections and tumor grade would significantly impact the assessment and prevention of brain tumors and provide a new avenue for novel therapeutic approaches.

## 5. Conclusions

Our findings show that infratentorial biopsy of brainstem and cerebellar lesions is a safe and effective way to acquire material for histological and molecular analyses, which is essential in the era of the CNS5 WHO 2021 classification. Moreover, patients with HGG were more frequently seropositive for *T. gondii* than patients with LGG. The results of our study support a hypothesis that *T. gondii* is associated with higher glioma grade through *EGFR* pathway activation. Lastly, MRI tumor enhancement should not be used to distinguish malignant from benign tumors.

## Figures and Tables

**Table 1:** Descriptive statistics of the performed biopsies.

		*N*	%
Gender		190	100.00%
	Female	96	50.50%
	Male	94	49.50%
Age (years), mean ± SD		41	16
KPS, mean ± SD		84	14
Diagnosis		190	100%
	Astrocytoma grade 2	68	35.80%
	Astrocytoma grade 3	37	19.50%
	Lymphoma	17	8.90%
	Glioblastoma	15	7.90%
	Non-neoplastic lesion	14	7.40%
	Metastatic tumor	10	5.30%
	Non-diagnostic biopsy	8	4.20%
	NA	5	2.60%
	Ependymoma	4	2.10%
	Pilocytic astrocytoma	4	2.10%
	Posterior fossa ependymoma grade 3	3	1.60%
	Oligodendroglioma grade 3	2	1.10%
	Medulloblastoma	2	1.10%
	Meningioma grade 1	1	0.50%
Side		117	100%
	Left	55	47.00%
	Right	50	42.70%
	Both	12	10.30%
Approach		145	100%
	Left	76	52.40%
	Right	69	47.60%
Symptoms		118	100.00%
	Blurred vision	49	41.50%
	Balance disorders	45	38.10%
	Paresthesia	37	31.30%
	Vertigo	33	27.90%
	Paresis	25	21.20%
	Speech disorders	21	17.80%
	Headache	14	11.80%
	Epilepsy	9	7.60%
	Dysphagia	7	5.90%
Multifocal tumors		15	100%
	Lymphoma	6	40%
	Astrocytoma grade 2	3	20%
	Astrocytoma grade 3	2	13.30%
	Glioblastoma	2	13.30%
	Oligodendroglioma grade 3	1	6.70%
	Benign lesion	1	6.70%
Complications			
	Without complications	179	94.50%
	Non-significant intracranial bleeding on CT	4	2.00%
	Pain during biopsy	2	1.00%
	Cerebrospinal fluid leak	1	0.50%
	Temporary ataxia	1	0.50%
	Worsening of paresis	1	0.50%
	CN VII palsy	1	0.50%
	Death	1	0.50%

Data are presented as *n* (%) unless otherwise stated. KPS—Karnofsky Performance Scale. SD—Standard Deviation. NA—not available. CT—computed tomography. CN VII—Cranial Nerve VII.

**Table 2:** Prevalence of tumors at various locations *.

Location		*N*	%
Brainstem		60	31.70%
	Pons	22	11.60%
	Brainstem	21	11.10%
	Pons + Medulla oblongata	10	5.30%
	Midbrain	3	1.60%
	Midbrain + Pons	2	1.10%
	Medulla oblongata	1	0.50%
	Midbrain + Pons + Medulla oblongata	1	0.50%
Cerebellum		62	32.70%
	Cerebellar peduncle	26	13.70%
	Cerebellar hemisphere	25	13.20%
	Cerebellar vermis	8	4.20%
	Cerebellar hemisphere + Cerebellar vermis	2	1.10%
	Cerebellar peduncle + Cerebellar vermis	1	0.50%
Brainstem + Cerebellum		46	24.30%
	Pons + Cerebellar peduncle	21	11.10%
	Pons + Medulla oblongata + Cerebellar peduncle	7	3.70%
	Pons + Cerebellar hemisphere	5	2.60%
	Pons + Cerebellar peduncle + Cerebellar hemisphere	4	2.10%
	Pons + Cerebellar vermis	3	1.60%
	Midbrain + Pons + Cerebellar peduncle	2	1.10%
	Pons + Cerebellar hemisphere + Cerebellar vermis	2	1.10%
	Medulla oblongata + Cerebellar peduncle	1	0.50%
	Pons + Cerebellar peduncle + Cerebellar vermis	1	0.50%
Area of 4th ventricle		22	11.60%
	Area of 4th ventricle	17	8.90%
	Cerebellar vermis + Area of 4th ventricle	3	1.60%
	Pons + Medulla oblongata + Area of 4th ventricle	2	1.10%

Note: *N* = 190; * Not possible to specify more precisely due to lack of data.

**Table 3:** Molecular diagnoses from infratentorial stereotactic biopsies.

	*IDH1* Mutation		*MGMT* Promoter Methylation		Codeletion of 1p19q	
Diagnosis	Wildtype	Mutant	Unmethylated	Methylated	Non-Codeleted	Codeleted
Astrocytoma grade 3	10	1	3	7	10	0
Astrocytoma grade 2	8	2	5	5	10	0
Glioblastoma	2	0	0	2	2	0
Pilocytic astrocytoma	1	0	1	0	1	0

Note: *IDH1*—isocitrate dehydrogenase 1; *MGMT*—O6-methylguanine-DNA methyltransferase.

**Table 4:** The relationship between the WHO grade, serum antibody status, and MRI contrast enhancement.

		Grade			Pearson Chi-Square			Phi Coefficient	
		LGG	HGG	Total	Value	df	*p*	Value	*p*
*Toxoplasma gondii*	Negative	20	6	26	14.679	1	<0.001 *	0.565	<0.001
	Positive	4	16	20					
	Total	24	22	46					
Epstein–Barr virus	Negative	2	3	5	0.402	1	0.526 a	−0.94	0.526
	Positive	22	18	40					
	Total	24	21	45					
MRI contrast enhancement	No	26	10	36	7.844	1	0.005 *	0.313	0.005
	Yes	18	26	44					
	Total	44	36	80					

Note: * The chi-square statistic is significant at the 0.05 level. a: More than 20% of cells in this subtable have expected cell counts less than 5. Chi-square results may be invalid. *N* = 46. LGG—Low grade glioma (WHO grade 1 and 2). HGG—High grade glioma (WHO grade 3 and 4). MRI—magnetic resonance imaging.

**Table 5:** Comparison of safety and efficacy between supratentorial and infratentorial approaches.

		Infratentorial		Supratentorial	
		*N*	%	*N*	%
Diagnostic material for histopathological evaluation		181/190	95.30%	61/63	96.80%
Complications					
	Without complications	179	94.50%	62	98.40%
	Non-significant intracranial bleeding on CT	4	2.00%		
	Pain during biopsy	2	1.00%	1	1.60%
	Cerebrospinal fluid leak	1	0.50%		
	Temporary ataxia	1	0.50%		
	Worsening of paresis	1	0.50%		
	CN VII palsy	1	0.50%		
Diagnostic material for molecular evaluation		26/29	89.70%	5/6	83.30%

CT—computed tomography. CN VII—Cranial Nerve VII.

## Data Availability

The data presented in this study are available on request from the corresponding author.

## Supplementary Materials

The following are available online at https://www.mdpi.com/article/10.3390/brainsci11111432/s1, Table S1: Relationship between age, gender, KPS, and diagnosis. Table S2. The relationship between serum antibody status, frequency of MRI contrast enhancement, and diagnosis. Table S3. Descriptive statistics of the patient group underwent supratentorial biopsy of cerebral peduncles lesions.

## References

[1] Laigle-Donadey F., Doz F., Delattre J.-Y. (2008). Brainstem gliomas in children and adults. Curr. Opin. Oncol..

[2] Rachinger W., Grau S., Holtmannspötter M., Herms J., Tonn J.-C., Kreth F.-W. (2009). Serial stereotactic biopsy of brainstem lesions in adults improves diagnostic accuracy compared with MRI only. J. Neurol. Neurosurg. Psychiatry.

[3] Weller M., van den Bent M., Preusser M., Le Rhun E., Tonn J.C., Minniti G., Bendszus M., Balana C., Chinot O., Dirven L. (2021). EANO guidelines on the diagnosis and treatment of diffuse gliomas of adulthood. Nat. Rev. Clin. Oncol..

[4] Reithmeier T., Lopez W.O., Doostkam S., Machein M., Pinsker M., Trippel M., Nikkhah G. (2013). Intraindividual comparison of histopathological diagnosis obtained by stereotactic serial biopsy to open surgical resection specimen in patients with intracranial tumours. Clin. Neurol. Neurosurg..

[5] WHO Classification of Tumours Editorial Board (2021). World Health Organization Classification of Tumours of the Central Nervous System.

[6] Kickingereder P., Willeit P., Simon T., Ruge M.I. (2013). Diagnostic Value and Safety of Stereotactic Biopsy for Brainstem Tumors: A Systematic Review and Meta-analysis of 1480 Cases. Neurosurgery.

[7] Roujeau T., Machado G., Garnett M.R., Miquel C., Puget S., Geoerger B., Grill J., Boddaert N., Di Rocco F., Zerah M. (2007). Stereotactic biopsy of diffuse pontine lesions in children. J. Neurosurg. Pediatr..

[8] Pincus D.W., Richter E.O., Yachnis A.T., Bennett J., Bhatti M.T., Smith A. (2006). Brainstem stereotactic biopsy sampling in children. J. Neurosurg. Pediatr..

[9] Śledzińska P., Bebyn M.G., Furtak J., Kowalewski J., Lewandowska M.A. (2021). Prognostic and Predictive Biomarkers in Gliomas. Int. J. Mol. Sci..

[10] McGirt M.J., Villavicencio A.T., Bulsara K.R., Friedman A.H. (2003). MRI-guided stereotactic biopsy in the diagnosis of glioma: Comparison of biopsy and surgical resection specimen. Surg. Neurol..

[11] Accuracy of Frameless and Frame-Based Image-Guided Stereotactic Brain Biopsy in the Diagnosis of Glioma: Comparison of Biopsy and Open Resection Specimen: Neurological Research. https://www.tandfonline.com/doi/abs/10.1179/016164105X40057.

[12] Kondziolka D., Lunsford L.D. (1995). Results and expectations with image-integrated brainstem stereotactic biopsy. Surg. Neurol..

[13] Chen S.-Y., Chen C.-H., Sun M.-H., Lee H.-T., Shen C.-C. (2011). Stereotactic biopsy for brainstem lesion: Comparison of approaches and reports of 10 cases. J. Chin. Med. Assoc..

[14] Dellaretti M., Reyns N., Touzet G., Dubois F., Gusmão S., Pereira J.L.B., Blond S. (2012). Stereotactic Biopsy for Brainstem Tumors: Comparison of Transcerebellar with Transfrontal Approach. Stereotact. Funct. Neurosurg..

[15] Kelly P.J., Gonçalves-Ferreira A.J., Herculano-Carvalho M., Pimentel J. (2003). Stereotactic biopsies of focal brainstem lesions. Surg. Neurol..

[16] Mathon B., Amelot A., Mokhtari K., Bielle F. (2019). Increasing the diagnostic yield of stereotactic brain biopsy using intraoperative histological smear. Clin. Neurol. Neurosurg..

[17] Sabbagh A.J., Alaqeel A.M. (2015). Focal brainstem gliomas. Neurosciences.

[18] Faulkner H., Arnaout O., Hoshide R., Young I.M., Yeung J.T., Sughrue M.E., Teo C. (2021). The Surgical Resection of Brainstem Glioma: Outcomes and Prognostic Factors. World Neurosurg..

[19] Cavalheiro S., Yagmurlu K., da Costa M.D.S., Nicácio J.M., Rodrigues T.P., Chaddad-Neto F., Rhoton A.L. (2015). Surgical approaches for brainstem tumors in pediatric patients. Childs Nerv. Syst..

[20] Schuman L.M., Choi N.W., Gullen W.H. (1967). Relationship of central nervous system neoplasms to *Toxoplasma gondii* infection. Am. J. Public Health Nations Health.

[21] John Steck M.D., William A.F.M.D. (1995). Stereotactic biopsy of brainstem mass lesions. Surg. Neurol..

[22] Samadani U., Judy K.D. (2003). Stereotactic Brainstem Biopsy Is Indicated for the Diagnosis of a Vast Array of Brainstem Pathology. Stereotact. Funct. Neurosurg..

[23] Jackson R.J., Fuller G.N., Abi-Said D., Lang F.F., Gokaslan Z.L., Shi W.M., Wildrick D.M., Sawaya R. (2001). Limitations of stereotactic biopsy in the initial management of gliomas. Neuro-oncology.

[24] Sanai N., Wachhorst S.P., Gupta N.M., McDermott M.W. (2008). Transcerebellar stereotactic biopsy for lesions of the brainstem and peduncles under local anesthesia. Neurosurgery.

[25] Tilgner J., Herr M., Ostertag C., Volk B. (2005). Validation of Intraoperative Diagnoses Using Smear Preparations from Stereotactic Brain Biopsies: Intraoperative versus Final Diagnosis—Influence of Clinical Factors. Neurosurgery.

[26] Furtak J., Mielczarek M., Szylberg M., Harat M. (2019). Biomarker concordance between molecular stereotactic biopsy and open surgical specimens in gliomas. Neurol. Neurochir. Pol..

[27] Ramakonar H.H. (2017). 220 A Stereotactic Brain Biopsy Needle Integrating an Optical Coherence Tomography (OCT) Probe with Blood Vessel Detection in Human Patients. Neurosurgery.

[28] Fritsch M.J., Leber M.J., Gossett L., Lulu B.A., Hamilton A.J. (1998). Stereotactic Biopsy of Intracranial Brain Lesions. Stereotact. Funct. Neurosurg..

[29] Fujimaki T., Hirata S., Terano N., Wakiya K., Adachi J.-I., Nishikawa R., Sasaki A., Kobayashi M. (2019). Surg-21. stereotactic biopsy for deep seated brain lesions using the leksell stereotactic frame system. Neuro-Oncology.

[30] Carruthers V.B., Suzuki Y. (2007). Effects of *Toxoplasma gondii* Infection on the Brain. Schizophr. Bull..

[31] Hodge J.M., Coghill A.E., Kim Y., Bender N., Smith-Warner S.A., Gapstur S., Teras L.R., Grimsrud T.K., Waterboer T., Egan K.M. (2021). *Toxoplasma gondii* infection and the risk of adult glioma in two prospective studies. Int. J. Cancer.

[32] Jung B.-K., Song H., Kim M.-J., Cho J., Shin E.-H., Chai J.-Y. (2016). High *Toxoplasma gondii* Seropositivity among Brain Tumor Patients in Korea. Korean J. Parasitol..

[33] Vittecoq M., Elguero E., Lafferty K.D., Roche B., Brodeur J., Gauthier-Clerc M., Missé D., Thomas F. (2012). Brain cancer mortality rates increase with *Toxoplasma gondii* seroprevalence in France. Infect. Genet. Evol..

[34] Ryan P., Hurley S.F., Johnson A.M., Salzberg M., Lee M.W., North J.B., McNeil J.J., McMichael A.J. (1993). Tumours of the brain and presence of antibodies to *Toxoplasma gondii*. Int. J. Epidemiol..

[35] Muniz-Feliciano L., Grol J.V., Portillo J.-A.C., Liew L., Liu B., Carlin C.R., Carruthers V., Matthews S., Subauste C.S. (2013). *Toxoplasma gondii*-Induced Activation of EGFR Prevents Autophagy Protein-Mediated Killing of the Parasite. PLoS Pathog..

[36] Portillo J.-A.C., Muniz-Feliciano L., Corcino Y.L., Lee S.J., Van Grol J., Parsons S.J., Schiemman W.P., Subauste C.S. (2017). *Toxoplasma gondii* induces FAK-Src-STAT3 signaling during infection of host cells that prevents parasite targeting by autophagy. PLoS Pathog..

[37] Corcino Y.L., Portillo J.-A.C., Subauste C.S. (2019). Epidermal growth factor receptor promotes cerebral and retinal invasion by *Toxoplasma gondii*. Sci. Rep..

[38] Ginsberg L.E., Fuller G.N., Hashmi M., Leeds N.E., Schomer D.F. (1998). The Significance of Lack of MR Contrast Enhancement of Supratentorial Brain Tumors in Adults: Histopathological Evaluation of a Series. Surg. Neurol..

[39] Pallud J., Capelle L., Taillandier L., Fontaine D., Mandonnet E., Guillevin R., Bauchet L., Peruzzi P., Laigle-Donadey F., Kujas M. (2009). Prognostic significance of imaging contrast enhancement for WHO grade II gliomas. Neuro-Oncology.

