# Supplementary material for: Infratentorial Stereotactic Biopsy of Brainstem and Cerebellar Lesions
Source: Brain Sci. 2021 Oct 28;11(11):1432. doi: 10.3390/brainsci11111432 (PMC8615913; doi:10.3390/brainsci11111432)
Supplement: Supplementary file 1 [file brainsci-11-01432-s001.zip › brainsci-1403119-supplementary.pdf]

Table S1. Relationship between age, gender, KPS, and diagnosis (N=190).

Diagnosis	Age		Karnofsky Performance Score		Gender			
					Female		Male	
	Mean	SD	Mean	SD	N	Row N %	N	Row N %
Astrocytoma grade 3	37	13	80	15	15	40.5%	22	59.5%
Posterior fossa ependymoma grade 3	31	9	65	7	1	33.3%	2	66.7%
Oligodendroglioma grade 3	33	1	85	7	2	100.0%	0	0.0%
Non-neoplastic lesion	44	17	86	16	9	64.3%	5	35.7%
Astrocytoma grade 2	35	15	88	13	31	45.6%	37	54.4%
Ependymoma	44	22	83	15	2	50.0%	2	50.0%
Glioblastoma	43	17	79	12	9	60.0%	6	40.0%
Lymphoma	59	12	84	14	12	70.6%	5	29.4%
Medulloblastoma	34	8	95	7	1	50.0%	1	50.0%
Metastatic tumor	62	9	77	11	3	30.0%	7	70.0%
Not available	45	12	83	17	1	20.0%	4	80.0%
Non-diagnostic biopsy	47	15	78	13	5	62.5%	3	37.5%
Pilocytic astrocytoma	24	7	95	7	4	1000%	0	0.0%
Meningioma grade 1	42	.	90	.	1	100.0%	0	0.0%

Note: N=190; SD - Standard Deviation.

Table S2. The relationship between serum antibody status, frequency of magnetic resonance imaging (MRI) contrast enhancement, and diagnosis.

	Epstein–Barr virus seropositivity				Toxoplasmosis seropositivity				Contrast enhancement			
	Negative		Positive		Negative		Positive		No		Yes	
Diagnosis	N	%	N	%	N	%	N	%	No	%	N	%
Astrocytoma grade 3	3	18.8%	13	81.3%	6	35.3%	11	64.7%	10	45.5%	12	54.5%
Non-neoplastic lesion	3	33.3%	6	66.7%	6	66.7%	3	33.3%	5	62.5%	3	37.5%
Astrocytoma grade 2	2	10.0%	18	90.0%	16	80.0%	4	20.0%	25	64.1%	14	35.9%
Ependymoma	0		2	100.0%	2	100.0%	0	0.0%	0	0.0%	3	100.0%
Glioblastoma	0		5	100.0%	0	0.0%	5	100.0%	0	0.0%	9	100.0%
Lymphoma	0		6	100.0%	3	50.0%	3	50.0%	1	9.1%	10	90.9%
Metastatic tumor	0		4	100.0%	0	0.0%	4	100.0%	0	0.0%	6	100.0%
Non-diagnostic biopsy	1	50.0%	1	50.0%	1	50.0%	1	50.0%	4	57.1%	3	42.9%
Pilocytic astrocytoma	0		2	100.0%	2	100.0%	0	0.0%	1	50.0%	1	50.0%

Note: $N_1 = 66; N_2 = 67, N_3=112$

Table S3. Descriptive statistics of the patient group underwent supratentorial biopsy of cerebral peduncles lesions.

		N	%
Sex			
	Female	23	36.5%
	Male	40	63.5%
Age, years (mean; SD)		51	16
KPS (mean; SD)		77	15.0%
Diagnosis			
	Astrocytoma grade 3	14	22.2%
	Glioblastoma	11	17.5%
	Lymphoma	11	17.5%
	Astrocytoma grade 2	9	14.3%
	Metastasis	9	14.3%
	Nondiagnostic	2	3.2%
	Oligoastrocytoma	2	3.2%
	Oligodendroglioma grade 3	1	1.6%
	Ependymoma	1	1.6%
	Ganglioglioma	1	1.6%
	Non neoplastic	1	1.6%
	Pilocytic Astrocytoma	1	1.6%
Multifocal tumors		4	100%
	Lymphoma	1	25%
	Astrocytoma grade 2	1	25%
	Glioblastoma	1	25%
	Metastasis	1	25%
Molecular evaluation*			
	IDH-mutant	1	20%
	IDH-wildtype	4	80%

MGMT methylated	4	80%
MGMT unmethylated	1	20%
1p/19q codeleted	1	20%
1p/19q noncodelated	4	80%

*Note: KPS - Karnofsky Performance Scale. SD - Standard Deviation. NA - not available. * - Biopsy material was subjected to molecular testing in 6 patients, which was non-diagnostic in one patient.*
